# Dietary Intake and Its Determinants Among Adults Living in the Metropolitan Area of Puerto Rico

**DOI:** 10.3390/nu11071598

**Published:** 2019-07-14

**Authors:** Josiemer Mattei, Martha Tamez, Sherman J. Bigornia, Sabrina E. Noel, Rui S. Xiao, Carlos F. Ríos-Bedoya, José F. Rodríguez-Orengo, Katherine L. Tucker

**Affiliations:** 1 Department of Nutrition, Harvard TH Chan School of Public Health, Boston, MA 02115, USA; 2 Department of Agriculture, Nutrition, and Food Systems; University of New Hampshire, Durham, NH 03824, USA; 3 Department of Biomedical and Nutritional Sciences, University of Massachusetts Lowell, Lowell, MA 01854, USA; 4 FDI Clinical Research of Puerto Rico, San Juan, PR 00927, USA; 5 McLaren Health Care, Grand Blanc, MI 48439, USA; 6 Department of Biochemistry, University of Puerto Rico Medical Sciences Campus, San Juan, PR 00936, USA

**Keywords:** food intake, nutrients, nutritional requirements, beverages and food, minority group, Hispanics, Latinos, Puerto Rico

## Abstract

There is scarce information regarding the dietary intake of adults living in Puerto Rico (PR). We aimed to assess intake of nutrients and foods, adherence to recommended intake of nutrients and diet quality, and sociodemographic and lifestyle factors correlated with diet quality among adults in the San Juan metropolitan area of PR. Data were obtained from participants of the cross-sectional convenience-sample Puerto Rico Assessment of Diet, Lifestyle, and Diseases (*n* = 248; ages 30–75 years). Diet quality was defined using the Alternate Healthy Eating Index 2010 (AHEI; range 0–110 indicating lower–higher quality). Linear regression models were used to relate AHEI to sociodemographic and lifestyle factors. Most participants met the Estimated Average Requirement (EAR) for iron, folate, and vitamins B12 and B6; 61% met the EAR for magnesium and 56% for calcium. Only 4% met the EAR for vitamin D, and 7% met the adequate intake for potassium. The main contributors to total energy intake were sugary beverages (11.8%), sweets/desserts (10.2%), dairy (8.5%), mixed dishes (7.6%), starches (6.3%), fast foods (5.5%), and rice (4.9%). The mean (SD) AHEI score was 59.8 (11.0). The lowest AHEI components for which recommended servings were met were red/processed meats, fruit, sodium, sugary beverages, and polyunsaturated fats, and the highest were nuts/legumes, omega-3 fats, and whole grains. Significantly higher AHEI scores were noted for older adults, other ethnicities (vs. Puerto Rican), being single, having some college or higher education, and never/formerly smoking. Adults living in PR report healthy and unhealthy dietary intakes, providing an opportunity to improve diet at the population level.

## 1. Introduction

Substantial evidence shows that diets rich in fruit and vegetables, dietary fiber, whole grains, and unsaturated fats, and low in sodium, sugars, and trans and saturated fat, can lower the risk of cardiovascular disease (CVD) and type 2 diabetes [1]. Not only is the assessment of single nutrients important, but also the assessment of overall diet quality, which has been linked to a lower risk of type 2 diabetes and CVD [2,3]. Thus, it is important to characterize the dietary intake and nutritional status of a population to prevent chronic disease.

Puerto Ricans living in the United States (US) have been shown to have a poor nutritional intake, as well as a low diet quality [4,5,6], especially when compared to other Hispanic/Latino heritages living in the US [7,8]. Consequently, these poor dietary habits have been associated with higher waist circumference, blood pressure, blood glucose, insulin, and inflammation, and lower HDL-C in this population [6,7,9]. The consistent associations among Puerto Ricans living in the US support crafting population-based nutritional messages and policies to improve dietary intake and prevent disease. Yet, it remains unclear whether these strategies would apply to residents of the country of origin—i.e., Puerto Rico (PR)—because little is known about the dietary intake of the population of PR. Cultural, socioeconomic, and environmental factors may drive differences in food choices and dietary intake among counterparts in a country of origin versus a host country [10], making it important to characterize the dietary intake of the population of PR.

The limited information on dietary intake available from PR suggests poor habits. Data from a cross-sectional study of adults residing in the San Juan metropolitan area showed a low intake of fruit and vegetables (1.9 servings/day) and a high intake of fried foods (2 servings/week) [11]. Other studies show a low intake of fruit, vegetables and dietary fiber, and a high intake of sugar-sweetened beverages [12,13]. Only 32% of adults in PR have been reported to have sufficient vitamin D levels [14]. Reports on food sources are more limited, but a recent small study among adult women residing in San Juan, PR showed that both traditional Puerto Rican foods such as legumes, rice, and plantains, as well as typical Western diet foods such as white bread and sweetened carbonated beverages, contributed to energy intake [15]. As of 2013, there were 59 fast-food establishments per 100,000 residents in PR, and these were visited 7.3 (men) and 6.4 (women) times/month [16]. Given these reports, it may be expected that poor diet quality was observed for 20% of a small study of health center patients [17].

The few existing reports of dietary intake from adults in PR have been conducted in small, geographically confined, or sex-specific studies, or have focused on selected foods or nutrients, with limited data on overall dietary quality. The lack of systematically analyzed dietary intake and nutritional status in PR continues to deter from establishing targeted nutritional messages for this population. Thus, we aimed to contribute new evidence by analyzing intake of multiple nutrients and foods, and adherence to recommended intake of nutrients, foods, and diet quality, as well as to determine the sociodemographic and lifestyle factors correlated with diet quality among adult men and women living in the metropolitan area of PR. A secondary aim was to analyze differences by sex and age groups in nutritional status and dietary intake. Based on reports among Puerto Ricans residing in the mainland US [18], we hypothesized that adults in PR would have a low intake of nutrients and low overall diet quality, and that nutritional status and diet quality would be better in women and in older adults.

## 2. Materials and Methods

### 2.1. Participants

Data were analyzed from the cross-sectional survey Puerto Rico Assessment of Diet, Lifestyle, and Diseases (PRADLAD). Details of the study recruitment and procedures have been previously described [19]. Briefly, between July and November 2015, a convenience sample of adults living in the San Juan, PR metropolitan area was recruited by advertising at three partner clinics: a research center and medical care facility, a primary care clinic within a city hospital, and a nonprofit federally-funded primary community health care center. Interested patients, their companions, or visitors to the clinics, were screened to confirm eligibility: (1) living in PR at the time of the study and for at least 10 months of the previous year, (2) aged 30–75 years, and (3) able to answer questions without assistance. All participants provided written informed consent. The Institutional Review Board (IRB) at Harvard T.H. Chan School of Public Health, Ponce Health Sciences University in PR, University of Massachusetts Lowell, and Northeastern University, approved the study in June 2015, with determinations of no greater than minimal risk to participants, and no vulnerable or special populations. This analysis adheres with the STrengthening the Reporting of OBservational studies in Epidemiology—Nutritional Epidemiology (STROBE-nut) guidelines [20].

### 2.2. Sociodemographic and Lifestyle Measures

Trained, bilingual research assistants administered all questionnaires in a private room at the clinics using the electronic data capture tool ‘Research Electronic Data Capture’ (REDCap) [21]. Information was collected on demographic characteristics, educational attainment, marital status, work history, household income, and food security and assistance through the Supplemental Nutrition Assistance Program (SNAP).

Participants were asked to report history, frequency, quantity, and type of smoking and alcohol use. Physical activity was captured using a modified Paffenbarger questionnaire of the Harvard Alumni Activity Survey [22], which was previously used in a Puerto Rican cohort [18]. A physical activity score was calculated as the sum of hours spent on typical activities over 24 h (heavy, moderate, light, or sedentary activity, and sleeping) multiplied by weighing factors that parallel the rate of oxygen consumption associated with each activity [18]. Sleep hours over 24 h and sleeping difficulties were assessed as previously described [23].

### 2.3. Dietary Assessment

Usual dietary intake over the past 12 months was self-reported using a semi-quantitative food frequency questionnaire (FFQ) adapted and validated for this population [24,25]. The FFQ was adapted from the National Cancer Institute-Block FFQ by including culturally-appropriate foods and portion sizes, and was shown to capture total nutrient estimates and ranking of individuals’ intake more accurately than the original questionnaire in a validation against 24-h recalls [24]. The FFQ has been validated against plasma carotenoids (correlations of 0.16 to 0.28) [26], vitamin E [27] and vitamin B12 [4] in older Hispanic adults. Participants were excluded if they had implausible energy intakes of <600 or >4800 kcal and/or 2 or more sections of the questionnaire left blank. The electronically-captured file was linked with the Minnesota Nutrient Data System (version 5.0_35) for food and nutrient analyses. Reported serving equivalents of individual foods were used to create food groups. Mixed dishes were disaggregated into individual food items then added to the appropriate food group. Dietary intake values are reported for dietary intake only, without supplement use.

Diet quality was measured using the Alternate Healthy Eating Index 2010 (AHEI), which has been shown to be associated with a lower risk of cardiometabolic disease [28], including among Puerto Ricans residing in the mainland US [6]. A score for each of 11 food groups or nutrient components was created by giving a continuous score between 0 for minimal observance of the recommended intake of the component and 10 points for maximal observance; intermediate values were prorated (i.e., the score for each AHEI food component ranged from 0 (least healthy) to 10 (healthiest)). The scores for all components were added, and the total AHEI score ranged from 0 (lowest diet quality) to 110 (highest diet quality).

### 2.4. Statistical Analyses

Of the 380 PRADLAD participants, 248 had complete and valid dietary data and were included in this analysis. Sociodemographic and behavioral characteristics were similar for PRADLAD participants with complete and valid FFQ data versus those who did not complete the FFQ, except that fewer participants with completed FFQs (vs. not) were unemployed (12.1% vs. 21.2%) and reported having sleep difficulties (45.5% vs. 61.0%) (Supplementary Table S1). Means (standard deviation) for nutrient intake, food group serving sizes, and AHEI (and its components) were calculated, and differences by sex and age categories were tested using *t*-test or ANOVA. All nutrients (except for percent from total energy intake of carbohydrates, protein, fat, and alcohol) were log-transformed to achieve normality, due to skewed distributions. Mean intake was adjusted for sex, age, and energy intake using the residuals method. For key micronutrients, intakes were estimated in relation to the age- and sex-specific Estimated Average Requirement (EAR), except for sodium and potassium for which there is no EAR and thus Adequate Intake (AI) was used instead, based on the current US government dietary reference intake tables. Differences by sex and age categories in the percent of participants meeting the EAR or AI were tested using chi-square or Fisher’s exact test. A sample size of 248 participants had sufficient power (>99%) to detect a medium effect size of 0.3 or large effect size of 0.5 in mean intake or in the proportion of individuals meeting recommended intake by sex or age category (assuming one or two degrees of freedom, respectively), using a two-sided significance level of 5% for a two-sample t-test or chi-square.

To determine factors associated with AHEI, we fitted a multivariable-adjusted general linear model including age, sex, ethnicity, marital status, educational attainment, household income, employment status, receiving SNAP, physical activity level, sleep hours, sleeping difficulties, smoking status, and total energy intake. Other factors, including food insecurity, living alone, household size, having health insurance, and rural residency, were tested in the model but made no change to the results. A sample size of 248 participants had sufficient power (>99%) to detect a mean difference (±SD) in AHEI of 1.0 (±2.5) units by exposure category, assuming a two-sided significance level of 5%. The proportional contribution (as percent) of food groups to total energy intake was estimated and then ranked from highest to lowest contribution. SAS version 9.4 (SAS Institute) was used for all analyses. All tests were 2-tailed; *p* < 0.05 was considered significant.

## 3. Results

### 3.1. Participant Characteristics

Most participants were women (69.0%), Puerto Rican (vs. other ethnicities, 79.4%), 43.7% were married or living with a partner, 62.4% had completed some college or higher education, 56.9% had a household income of $10,000 or less, 47.6% were retired or stay-at-home, and 50.4% were SNAP recipients (Supplementary Table S2). Among healthy lifestyle behaviors, 28.8% reported moderate/vigorous physical activity, 53.8% slept 7–8 h/day, 54.5% rarely had sleeping difficulties, and 82.6% never or formerly smoked.

### 3.2. Nutrient and Food Intake

Carbohydrate contributed to 53.4% of total energy intake, followed by dietary fats (30.9%), which were mostly monounsaturated (MUFA) and saturated (SFA) fatty acids (Table 1). Mean (SD) animal protein intake was higher than plant protein intake (51.3 (16.0) vs. 27.2 (6.6) g/day). Mean intake was generally similar by sex, except for a higher intake of total energy (2388 (109) vs. 2117 (75) kcal/day) and a lower intake of iron (14.6 (0.4) vs. 15.6 (0.3) mg/day) for men vs. women. Significantly higher mean intakes of dietary fiber, magnesium, and potassium, but lower intakes of starch-to-total-fiber ratio and vitamin B12 were observed with increasing age category.

Few participants met the age- and sex-specific dietary recommended intake of vitamin D (3.6%) or potassium (6.9%), while more than half of the participants met the recommendations for calcium (55.7%) and magnesium (60.5%), most met them for folate (90.3%) and vitamins B12 (94.8%) and B6 (99.2%), and everyone (100%) met the recommended intake for iron (Table 2). Significantly fewer men than women met the EAR for magnesium (19.5% vs. 79.9%, *p* < 0.001). Fewer older adults (61–75 years) than younger adults (30–45 years) met the EAR for vitamin B12 (92.7% vs. 98.5%) and calcium (47.3% vs. 70.6%), but more met the recommended intake for potassium (14.6% vs. 0%). No adults aged 30–45 years met the AI for potassium.

Ranking foods by percent contribution to total energy showed that the top food groups were sugary beverages (11.8% of daily energy intake), sweets and desserts (10.2%), dairy (8.5%), mixed dishes and soups (7.6%), starchy vegetables (6.3%), fast foods (5.5%), and rice (4.9%) (Table 3). The rice category excluded some rice intake that was instead included in the mixed dishes group as mixed riced-based dishes (i.e., 19.3% of the total rice intake, or 41.6 g/day, data not shown). Within sugary beverages, juices contributed more to energy than sodas and other sugary drinks (7.9% vs. 3.9%). The main sources of starchy vegetables were potatoes, plantains, and sweet potatoes. Animal products included processed meats (3.6% of daily energy intake), eggs (3.6%), poultry (2.9%), fish and seafood (2.3%), and red meat (2.2%). Water does not contribute energy, but mean intake (SD) was 3.9 (3.4) servings/day. Similarly, spices and condiments contributed little to energy intake but 3.0 (2.3) servings/day were consumed, on average. The ranking of the top energy-contributing food groups was similar by sex, except for a higher intake (servings/day) of eggs among men than women (0.84 (1.03) vs. 0.58 (0.66)) (Supplementary Table S3). Daily servings of several food groups differed by age group.

### 3.3. Diet Quality

**Figure 1:**
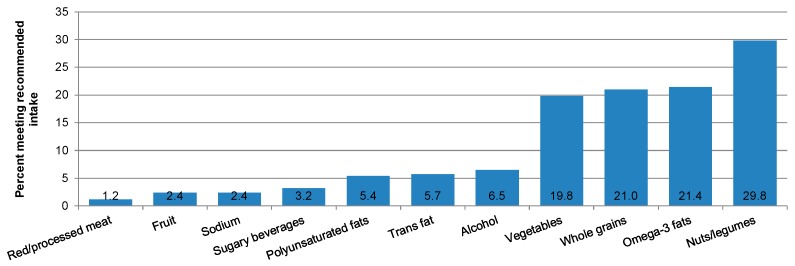
Percent of adults living in Puerto Rico meeting the recommended intake of food and nutrient components of the Alternate Healthy Eating Index.

The total mean (SD) AHEI score was 59.8 (11.0), with no differences by sex or age category (Table 4). For AHEI individual components, higher mean scores were observed for trans fats (7.9 (1.3)) and omega-3 fatty acids (7.2 (3.3)), and lowest mean scores were observed for whole fruit (2.9 (2.6)) and sugary beverages and fruit juice (1.4 (2.6)). Men had a higher score for the alcohol component than women (7.2 (1.7) vs. 5.8 (6.1), *p* < 0.001). A significantly higher mean intake of vegetables, whole fruit, and whole grains, and a higher mean score of whole fruit and red/processed meats (lower intake) was observed by increasing age category. A lower mean intake of omega-3 fatty acids in the 61–75 years age category (261 (250) mg/day) vs. the 46–60 year (392 (396) mg/day) and 30–45 year (274 (222) mg/day) age categories was noted. Few participants met the recommended servings/day for red/processed meats (1.2%), whole fruit (2.4%) or sugary beverages and fruit juice (3.2%); the most commonly met components were nuts/legumes (29.8%), omega-3 fatty acids (21.4%), whole grains (21.0%), and vegetables (19.8%) (Figure 1).

The recommended amounts are: ≥5 serving/day of vegetables without potatoes; ≥4 serving/day of fruit without fruit juices; ≥½ cup of whole grains; <1 serving/day of sugary beverages; ≥1 serving/day of nuts/legumes; <1.5 serving/day red/processed meats; ≤0.5% energy from trans fats; >250 mg/day omega-3 fatty acids; ≥10% energy from polyunsaturated fatty acids (PUFA); <2300 mg/day of sodium; ≤2 drinks/day for men or ≤1drink/day for women of alcohol.

For AHEI components, positive significant correlations were noted for intake of fruit with vegetables and whole grains; vegetables with nuts/legumes, whole grains, and sodium; red and processed meats with nuts/legumes, sugary beverages, omega-3 fatty acids, trans fats, sodium, and alcohol; nuts/legumes with sugary beverages, whole grains, polyunsaturated fatty acids (PUFA), and sodium; sugary beverages with sodium; whole grains with sodium; omega-3 fatty acids with PUFA, trans fat, and sodium; PUFA with trans fat and sodium; trans fat with sodium; and sodium with alcohol. Inverse significant correlations were observed for intake of trans fat with fruit, vegetables, nuts/legumes, sugary beverages, and whole grains; and PUFA with alcohol (data not shown).

### 3.4. Determinants of Diet Quality

**Figure 2:**
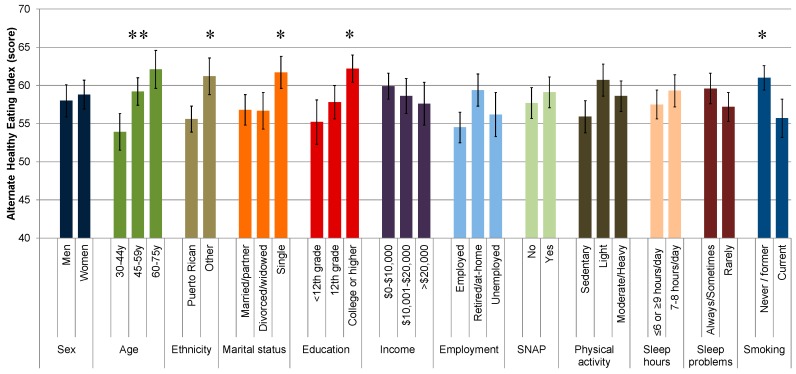
Mean (standard error) of the Alternate Healthy Eating Index by sociodemographic and lifestyle factors in adults living in Puerto Rico. Shown as mean (standard error) from a multivariable-adjusted general linear model including all variables shown and total energy intake. AHEI score is a range of 0–110 points with higher scores indicative of better diet quality. Significant factors shown as * *p* < 0.05; ** *p* < 0.01. *n* = 248.

A multivariable-adjusted general linear model showed that age, ethnicity, marital status, educational attainment, and smoking status were significantly associated with AHEI; physical activity level was marginally significant (Figure 2; Supplementary Table S2). Mean (SD) AHEI was higher among older rather than younger adults (62.1 (2.5) vs. 59.2 (1.8) vs. 53.9 (2.4), *p* = 0.009); ethnicities other than Puerto Rican (61.2 (2.4) vs. 55.6 (1.7), *p* = 0.011); single rather than married or divorced adults (61.7 (2.1) vs. 56.7 (2.4) vs. 56.8 (2.0), *p* = 0.037); adults with some college or higher education vs. 12th grade or less (62.2 (1.8) vs. 57.8 (2.2) vs. 55.2 (2.9), *p* = 0.026); and never/former smokers rather than current smokers (61.0 (1.6) vs. 55.7 (2.5), *p* = 0.018).

## 4. Discussion

Adults residing in the metropolitan area of PR have mostly unhealthy nutritional status and dietary quality, evidenced by highly-ranked unhealthy foods (such as sweets, sugary beverages, and fast foods) and low adherence to the recommended intakes of potassium, vitamin D, and high-quality food groups. However, some nutrients and foods were observed at healthy recommended intakes, such as B vitamins, nuts and legumes, trans fat, and omega-3 fatty acids. Sociodemographic factors associated with better diet quality included older age, non-Puerto Rican ethnicity, single status, and higher educational attainment. The observations emphasize both nutrients and foods as well as population subgroups that should be targeted to improve dietary intake in PR with the goal of ultimately reducing the risk of chronic disease.

Similar to our study, others have found consistent results for low intake of fruit and vegetables and high intake of fried foods, starches, rice, and sugar-sweetened beverages [12,13,15]. A recent study ranked the top foods contributing to total energy among women in PR as white bread/rolls/crackers, rice, plantain dishes, rice and vegetable dishes, and reduced-fat milk [15]. While the percent contribution and specific ranking varied from our study, likely due to differences in food groupings, the results were consistent in ranking refined carbohydrates, dairy, and starch as the main contributors to energy. For example, the total intake of rice is likely higher than reported in our sample as some rice intake was instead captured in the mixed dishes group. Because the proportion of rice to mixed dishes was small, we expect that disaggregating the components would have changed the food rankings just slightly. The top foods consumed by adults in our study—in some cases contributing a large proportion of energy—are of clinical concern for the population’s health, given that intake of sugary beverages (including commercial juices) [29,30], sweets and desserts [31], starchy foods [32], fast foods [33], and white rice [34,35] has been associated with a higher risk of cardiometabolic conditions. Furthermore, adults in this sample in PR consumed mostly animal protein rather than plant-based protein such as nuts and legumes, and animal protein has been linked to higher cardiovascular mortality [36]. Animal products were mostly comprised of processed meats, also associated with a higher risk of chronic disease [37]. Healthy foods that ranked lower in our sample and that should be emphasized in nutritional programs include water, spices and condiments, whole grains, nuts, legumes, fish and seafood, and fruit.

We showed extremely low adherence to sex- and age-specific recommendations of vitamin D and potassium, in agreement with previous work in PR [14] and with current estimates in the general US population [38,39]. While we noted vitamin D adequacy in 3.6% of our sample using dietary intake only, a study in PR that measured serum vitamin D accounting for sunlight sources showed that only 31.5% of the studied population had sufficient vitamin D concentrations, suggesting that vitamin D deficiency may be prevalent even in areas rich in sunlight such as PR [14]. Vitamin D sources include eggs and fortified dairy; the former did not rank highly in this population and the latter ranked highly, but we could not assess information on fortification. The low consumption of fruits and vegetables likely explains the low adherence to potassium and magnesium recommendations. Despite relatively low intakes of red meat and seafood, iron requirements were generally met, likely from the intake of legumes and some fortified cereals. Strategies to improve nutritional status in PR should include policies for vitamin D fortification [40], clinically-managed vitamin D supplementation [41], and public health programs that promote fruit and vegetable consumption. Additionally, public health messages should be tailored by age groups, with emphasis on increasing dietary fiber, magnesium, and potassium among younger adults, and vitamin B12 and calcium among older adults.

The diet quality scores for components of the AHEI were mixed, with some components scoring higher, such as trans fat and omega-3 fatty acids, and some scoring poorly, such as whole fruit and sugary beverages. Despite the high intake of sweets and desserts and fast foods, PR has had mandatory measures on trans fats and banned its use in restaurants since 2007 [42,43], likely explaining the higher scoring for this component. Plant oils (such as canola oil) and fortified foods may contribute to omega-3 fatty acid intake, as intake of fish and nuts was low. Of note, the low intake of omega-3 fatty acids in the 61–75 years age category should be addressed, as this has been associated with a higher risk of cognitive decline and metabolic syndrome in Puerto Ricans [44,45]. Concurrent with the food and nutrient results, adherence to recommended servings/day for diet quality components was poor for red/processed meats (due mainly to processed meats), fruit, and sugary beverages. Furthermore, poorer diet quality was noted among younger adults, reinforcing the priority for nutritional programs for this age group.

Improvements in diet quality should be emphasized among participants of Puerto Rican ethnicity, married or divorced adults, adults with an educational attainment of high school or lower, and current smokers. Similar correlations between sociodemographic factors and diet in studies in PR have been reported. Among adults residing in San Juan, a higher consumption of fruit and vegetables was observed among older adults and those with higher educational levels, whereas higher intakes of fast food and fried foods were noted for younger ages, higher income, and 12–15 years of formal education [11]. Our study included participants of other ethnic groups, predominantly Dominican or US-American, and studies concur that Puerto Ricans in the mainland US report lower diet quality and nutritional intake than other ethnic groups [7,8]. Both education and income have been correlated with diet quality in the general US population [46], but in our study, education took a more prominent role. The economic downturn in PR [47] may have attenuated the impact of income on food choices, as people cut back expenditures or shift their diets toward staple foods and less expensive unhealthy foods [48].

Our results mirror the poor dietary behaviors and nutritional intake reported for Puerto Ricans living in the US, who have been shown to have low intakes of vitamin C, dietary fiber, fruit, vegetables, whole grains, fish, and high intakes of saturated and trans fat, added sugars, sodium, sugary beverages and red/processed meats [5,7,8]. Surprisingly, diet quality as measured using the AHEI was higher in our sample (59.8 (11.0)) than for Puerto Ricans aged 45–75 years living in Boston (54.0 (8.9)) and for Puerto Ricans aged 18–74 years living in four US cities (43.0 (0.2)). This may be explained by differences in the cohorts; for example, the Boston-Puerto Rican cohort was mostly low income with lower educational attainment and multiple chronic conditions [18], and the US-based Puerto Rican study was restricted to participants without diabetes, who tended to have a better diet, and included younger participants, who tended to have a more inadequate diet. Other factors, such as food access and acculturation, may also drive differences in food choice between groups [10]. We have shown that Puerto Ricans in the US mainland with stronger ethnic orientation had poorer AHEI, but higher adherence to a traditional diet marked by rice, beans, and oils [49]. The combined results underscore that Puerto Ricans in both PR and the mainland US need to improve their diets, and that dietary intake should be studied for this ethnic group distinctly in the mainland US and in their country of origin.

Our study is limited by its cross-sectional design, limiting our ability to establish directionality of the factors associated with diet quality. PRADLAD showed wide socioeconomic representation across clinics [19], but the convenience recruitment in the metropolitan area of PR only may limit generalizability. Specifically, our sample was predominantly comprised of adults with college or higher education, and fewer participants with complete FFQs were unemployed, which may generate selection bias by socioeconomic status. Limited sample size may have reduced our ability to detect some potentially significant correlates of diet quality, such as rural residency (16% of our sample) and frequent food insufficiency (15%) [50], and future larger studies should determine these associations. Furthermore, other characteristics not tested in our study may explain dietary intake, such as seeking information on health or medical topics from health care providers [51], or awareness of current recommendations [12]. Finally, our study was conducted in 2015 and it will be important that future studies recognize recent nutritional status in PR after the impact of Hurricanes Irma and Maria in 2017, as other studies have shown changes in food-related behaviors post-disaster [52,53]. Our study is strengthened by the use of a validated dietary assessment tool and a comprehensive food and nutrient composition database that captured the traditional intake of adults in PR.

## 5. Conclusions

We characterized the dietary intake and nutritional status, as well as the sociodemographic factors correlated with diet quality, of adults living in the metropolitan area of PR. Furthermore, we identified foods and nutrients that should be prioritized in dietary programs and initiatives according to age categories, with younger adults generally having poorer diet quality and nutritional status but older adults needing to improve their intakes of vitamin B12, calcium, and omega-3 fatty acids. Implications for public health include the need to strengthen strategies for dietary improvements at the population level, which may include couple- or family-based programs [54] and enhancing nutrition and health literacy [55]. Clinically, nutrition and health professionals in PR should be aware of the foods and nutrients that are challenging for their patients’ diet and which subgroups are more vulnerable, so that they can tailor counseling. We have previously shown in this cohort that participants who self-rated their diet quality as fair/poor (vs. excellent/very good) had twice the odds of having two or more chronic diseases [50]. Future research, especially observational studies with large sample size, should expand dietary assessment to the whole territory of PR and to wider age ranges, for a representative evaluation; should assess biomarkers of intake; and should update the analysis to reflect the situation post-Hurricane Maria. Dietary interventions and implementation-science studies aiming to improve nutritional intake and promote healthy diets in PR should also be conducted. Additionally, to better understand the sociodemographic drivers of food choices and diet quality in PR exposed in our study, researchers and public health stakeholders should investigate factors across the socioecological model, such as cost and availability of healthy vs. unhealthy foods, incentives and tax policies, government-sponsored programs, and educational opportunities across the life stages [56]. Thus, to ultimately reduce the risk of chronic disease at the population level, our study supports the public health, clinical, and research need to improve diet among adults living in PR, who report some healthy, but many unhealthy dietary behaviors.

## Figures and Tables

**Table 1:** Mean intakes of energy and nutrients of adults living in Puerto Rico, by age and sex category.

Dietary Intake ^a^	Overall ^b^	Sex ^c^		Age ^d^		
	(*n* = 248)	Men (*n* = 77)	Women (*n* = 171)	30–45 y (*n* = 68)	46–60 y (*n* = 125)	61–75 y (*n* = 55)
Total energy, kcal/d	2228 (940)	2388 (109) *	2117 (75)	2440 (118)	2293 (84)	2026 (132)
Percent from total energy						
Carbohydrate %	53.4 (7.4)	54.0 (0.9)	53.7 (0.6)	53.4 (0.9)	52.7 (0.7)	55.5 (1.1)
Protein %	15.1 (2.8)	14.6 (0.3)	15.1 (0.2)	14.8 (0.3)	15.3 (0.2)	14.5 (0.4)
Fat %	30.9 (5.9)	30.5 (0.7)	30.7 (0.5)	30.8 (0.7)	31.3 (0.5)	29.7 (0.8)
Alcohol % ^e^	0.7 (2.1)	1.0 (0.3)	0.5 (0.2)	1.0 (0.3)	0.7 (0.2)	0.5 (0.3)
Total carbohydrate, g/d	299 (43)	302 (5.0)	301 (3.5)	301 (5.4)	296 (3.9)	308 (6.1)
Dietary fiber	24.1 (7.7)	24.1 (0.9)	24.5 (0.6)	22.0 (0.9) **	23.9 (0.7)	27.2 (1.1)
Starch	106 (25)	107 (3)	107 (2)	108 (3)	103 (2)	109(4)
Added sugar	75.0 (35.8)	76.5 (4.2)	74.8 (2.9)	79.4 (4.6)	73.9 (3.3)	73.7 (5.1)
Starch to total fiber ratio	4.7 (1.6)	4.7 (0.2)	4.7 (0.1)	5.2 (0.2) **	4.6 (0.1)	4.3 (0.2)
Total protein, g/d	85.5 (16.2)	83.5 (1.9)	85.1 (1.3)	84.0 (2.0)	87.0 (1.5)	82.0 (2.3)
Vegetable protein	27.2 (6.6)	26.9 (0.8)	27.6 (0.5)	26.8 (0.8)	26.6 (0.6)	28.4 (0.9)
Animal protein	51.3 (16.0)	49.0 (1.9)	51.0 (1.3)	49.8 (2.0)	53.1 (1.4)	47.0 (2.3)
Total fat, g/d	79.5 (14.5)	78.1 (1.7)	79.7 (1.2)	78.0 (1.8)	80.2 (1.3)	78.5 (2.1)
Monounsaturated fatty acids	26.3 (5.4)	25.6 (0.6)	26.4 (0.4)	25.3 (0.7)	26.6 (0.5)	26.2 (0.8)
Polyunsaturated fatty acids	15.8 (4.4)	15.5 (0.5)	15.8 (0.4)	16.0 (0.6)	15.9 (0.4)	15.1 (0.6)
Omega-3 fatty acids	1.7 (0.6)	1.7 (0.1)	1.7 (0.0)	1.7 (0.1)	1.8 (0.1)	1.6 (0.1)
Saturated fatty acids	23.0 (5.7)	22.5 (0.7)	23.3 (0.5)	22.6 (0.7)	23.2 (0.5)	22.6 (0.8)
Trans fatty acids	2.8 (1.0)	2.7 (0.1)	2.7 (0.1)	2.8 (0.1)	2.8 (0.1)	2.5 (0.1)
Cholesterol, mg/d	355.7 (202.3)	366.7 (23.7)	339.7 (16.3)	335.5 (25.6)	380.7 (18.3)	343.3 (28.8)
Total alcohol ^e^, g/d	2.6 (10.7)	4.2 (1.3)	1.7 (0.9)	4.1 (1.4)	3.1 (1.0)	1.7 (1.7)
Caffeine, g/d	132.4 (138.9)	140.0 (16.4)	131.6 (11.3)	128.7 (17.9)	131.6 (12.7)	147.0 (19.9)
Vitamin D, mcg/d	4.7 (2.6)	4.1 (0.3)	4.8 (0.2)	4.1 (0.3)	4.9 (0.2)	4.3 (0.4)
Vitamin B12, mcg/d	9.7 (8.2)	8.8 (1.0)	9.4 (0.7)	10.2 (1.0) *	10.3 (0.7)	7.0 (1.2)
Vitamin B6, mg/d	2.3 (1.1)	2.4 (0.1)	2.3 (0.1)	2.2 (0.1)	2.4 (0.1)	2.4 (0.2)
Folate, mcg/d	453 (128)	440 (15)	461 (10)	441 (16)	449 (12)	462 (18)
Calcium, mg/d	954 (295)	911 (35)	973 (24)	912 (37)	951 (27)	963 (42)
Magnesium, mg/d	317 (70)	309 (8)	322 (6)	296 (9) *	316 (6)	334 (10)
Iron, mg/d	15.5 (3.5)	14.6 (0.4) *	15.6 (0.3)	15.3 (0.4)	15.5 (0.3)	14.6 (0.5)
Sodium, mg/d	3754 (790)	3701 (93)	3762 (64)	3780 (100)	3752 (72)	3665 (113)
Potassium, mg/d	3366 (810)	3388 (93)	3399 (64)	3149 (101) **	3356 (72)	3675 (114)

Shown as mean (SD). Significant differences determined from t-test or ANOVA shown as * *p* < 0.05 or ** *p* < 0.01. ^a^ Dietary intake values correspond to dietary intake only (without supplements use) in grams per day unless indicated otherwise. All nutrients (except for percentages from total energy intake of carbohydrates, protein, fat, and alcohol) were log-transformed to restore normality due to skewed distributions. ^b^ Adjusted for age, sex, and energy intake using the residuals method, except for total energy intake. ^c^ Adjusted for age and energy intake using the residuals method, except for total energy intake which was only adjusted for age. ^d^ Adjusted for sex and energy intake using the residuals method, except for total energy intake which was only adjusted for sex. ^e^ Alcohol intake was restricted to only those who reported consuming alcoholic beverages (*n* = 234). y: years; d: day.

**Table 2:** Percent of adults living in Puerto Rico meeting the age- and sex-specific dietary recommended intake of micronutrients.

Dietary Intake ^a^	Overall	Sex		Age		
	(*n* = 248)	Men (*n* = 77)	Women (*n* = 171)	30–45 y (*n* = 68)	46–60 y (*n* = 125)	61–75 y (*n* = 55)
Vitamin D, mcg/d	3.6	1.3	4.7	1.5	6.4	0
Vitamin B12, mcg/d	94.8	96.1	98.3	98.5	99.2	92.7 *
Vitamin B6, mg/d	99.2	97.4	100	100	98.4	100
Folate, mcg/d	90.3	88.3	91.2	92.7	92.0	83.6
Calcium, mg/d	55.7	62.3	52.6	70.6	51.2	47.3 *
Magnesium, mg/d	60.5	19.5	79.9 ***	58.8	59.2	65.5
Potassium, mg/d	6.9	7.8	6.4	0	7.2	14.6 **

Significant differences determined from chi-square or Fisher’s exact-test shown as * *p* < 0.05; ** *p* < 0.01; *** *p* < 0.001; ^a^ Shown as percent meeting the Estimated Average Requirement (ERA, or the value estimated to meet the requirement of half the healthy individuals in the group), except for sodium and potassium, for which there is no ERA and Adequate Intake (AI, or the value based on observed or experimentally determined approximations of nutrient intake by a group of healthy people), which were estimated instead. Corresponds to dietary intake only (without supplements use) adjusted for energy intake using the residuals method. Iron and sodium are not shown as these met or exceeded the recommendations (i.e., met by 100% of the population). y: years; d: day.

**Table 3:** Food ranking by percent contribution to total energy reported by adults living in Puerto Rico.

Food Group	% of Daily Energy Intake ^a^	Servings/Day ^a^	Sample Foods Included in the Food Group Category
All sugary beverages	11.79 (9.43)	2.32 (2.13)	Includes juices, sodas and other sugary beverages
Juices	7.93 (8.10)	1.48 (1.62)	Fruit drinks (Sunny Delight, Kool-Aid), fruit juices, tomato or vegetable juice, and nectars
Sodas and other sugary beverages	3.85 (4.71)	0.84 (1.11)	Presweetened coffee or tea, sodas (cola, 7-up, ginger ale), sport drinks (Gatorade, Powerade), energy drinks (Red Bull), chocolate milk (whole, fat free, low fat, soy)
Sweets and desserts	10.21 (6.79)	3.89 (2.99)	Cakes, brownies, pies, sorbets, ice cream, candy, chocolate, cookies, honey, jelly
Dairy	8.49 (6.23)	1.82 (1.54)	Cheese, sour cream, milk (soy, low fat, whole, nonfat), yogurt (whole, nonfat, low fat)
Mixed dishes	7.62 (5.83)	0.87 (0.83)	Mixed dishes and soups
Mixed dishes	5.40 (4.07)	0.43 (0.38)	Enchiladas, tamales, quesadillas, burritos, pot pies, empanadas, rice mixed dishes
Soups	2.22 (3.21)	0.44 (0.58)	Vegetables soups, clam chowder, chicken or beef broths
Starchy vegetables	6.25 (6.29)	1.10 (1.38)	Plantains, potatoes, and sweet potatoes
Potatoes	3.69 (4.38)	0.71 (1.01)	Baked or boiled potatoes, mashed potatoes; excluding French fries
Plantains	1.42 (1.92)	0.14 (0.18)	Plantains and green bananas, including pasteles
Sweet potato	1.14 (1.82)	0.25 (0.42)	Boiled or baked sweet potatoes
Fast foods	5.50 (5.02)	0.44 (0.47)	Hamburgers (Burger King, McDonalds), French fries, pizza (Pizza Hut), tacos (Taco Bell)
Rice	4.86 (4.26)	0.53 (0.49)	Brown and white rice
Fats	4.15 (2.52)	0.87 (0.57)	Butter, margarine, lard, mayonnaise, and dressings
Refined grains	4.13 (3.26)	0.57 (0.47)	Ready-to-eat cereals (e.g., Froot Loops, Cheerios, Corn Pops, Corn Flakes), grits, white bread, flour tortilla, bagels, waffles, pancakes, cornbread
Fruit	3.85 (3.90)	1.13 (1.08)	Fresh, frozen, dried, and canned fruits
All vegetables	3.81 (3.29)	3.34 (2.56)	Yellow, green, and other vegetables, tomatoes
Yellow vegetables	0.59 (1.05)	0.31 (0.43)	Raw, cooked, and canned yellow vegetables (e.g., carrots, pumpkin, squash)
Green vegetables	0.40 (0.56)	0.40 (0.46)	Raw, cooked, and canned dark-green vegetables (e.g., broccoli, spinach, romaine, collards)
Tomatoes	0.35 (0.33)	0.44 (0.42)	Raw, cooked, and canned tomato, tomato sauce, tomato puree, tomato paste, tomato-based sauce, excluding ketchup
Other vegetables	2.46 (2.14)	2.20 (1.79)	Raw, cooked, and canned vegetables (e.g., corn, peppers, cucumber, radish, onion, mushrooms, eggplant)
Processed meats	3.64 (3.10)	0.72 (0.82)	Lunchmeats, sausages, ham, bacon
Eggs	3.59 (3.94)	0.66 (0.80)	Boiled, scrambled, and whole eggs, including egg substitutes (e.g., Egg Beaters)
Whole grains	3.54 (3.09)	0.53 (0.47)	Corn tortilla, oats, whole wheat bread, whole wheat pasta, whole grain cereals (100% Bran, Oat Bran Flakes, All-Bran Wheat Flakes, granola)
Legumes	3.14 (2.81)	0.63 (0.58)	Pinto, black, kidney, lima beans, chickpeas, green peas
Poultry	2.92 (2.68)	0.32 (0.28)	Turkey, chicken (breast, leg)
Fish and seafood	2.28 (2.95)	0.37 (0.48)	Cod, salmon, sardines, haddock, tuna, catfish (cooked from fresh or frozen), clams, crab, scallops, lobster, oyster
Snacks	2.25 (2.83)	0.41 (0.70)	Crackers, popcorn, chips, pretzels
Red meats	2.22 (2.27)	0.28 (0.35)	Beef of ground beef, pork, lamb, organs
Pasta	2.12 (2.53)	0.21 (0.24)	Lasagna, spaghetti, noodles
Oils	2.06 (2.08)	0.34 (0.34)	Canola, corn, olive, sesame, or soybean oils
Nuts	1.31 (2.40)	0.23 (0.46)	Almonds, cashews, peanuts, pecans, pine nuts, sunflower seeds, walnuts, pistachios, peanut butter
Alcohol	0.86 (2.49)	0.24 (0.89)	Beer, liquor, wine
Artificial-sweetened beverages	0.80 (3.09)	0.13 (0.43)	Diet sodas, low-calorie milkshakes (e.g., Slim-Fast)
Spices and condiments	0.55 (0.55)	3.03 (2.29)	Pickles, horseradish, hot pepper, hot sauce, vinegar, oregano, paprika, turmeric, cumin, Italian seasoning, chili powder, cinnamon, mustard
Unsweetened beverages	0.17 (0.19)	1.41 (1.49)	Unsweetened tea, coffee
Salt	0.00 (0.00)	0.47 (0.41)	Salt
Water	0.00 (0.00)	3.90 (3.40)	Bottled or tap water, sparkling water

^a^ Shown as unadjusted mean (SD). Estimated with SAS PROC RANK.

**Table 4:** Mean (standard deviation) of the Alternate Healthy Eating Index and its components in adults living in Puerto Rico.

	Overall ^a^	Sex ^a^		Age ^a^		
	*n* = 248	Men (*n* = 77)	Women (*n* = 171)	30–45 y (*n* = 68)	46–60 y (*n* = 125)	61–75 y (*n* = 55)
Total AHEI score	59.8 (11.0)	59.4 (10.7)	60.0 (11.2)	58.0 (11.0)	59.7 (11.0)	62.3 (10.9)
Vegetable score	6.3 (2.9)	6.4 (2.9)	6.2 (3.0)	5.8 (2.7)	6.3 (2.9)	6.9 (3.2)
Whole fruit score	2.9 (2.6)	2.8 (2.2)	3.0 (2.6)	2.2 (2.2)	2.9 (2.4)	3.9 (2.3) **
Whole grains score	5.3 (3.5)	4.7 (3.6)	5.5 (3.4)	4.8 (3.5)	5.3 (3.5)	5.8 (3.5)
Sugary beverages and fruit juice score	1.4 (2.6)	1.3 (2.6)	1.4 (2.7)	1.5 (2.9)	1.2 (2.4)	1.7 (2.9)
Nuts and legumes score	6.4 (3.2)	6.5 (3.0)	6.3 (3.3)	6.8 (2.8)	6.2 (3.3)	6.2 (3.4)
Red/processed meat score	4.9 (3.5)	4.6 (3.7)	5.0 (3.4)	4.0 (3.4)	4.9 (3.5)	6.0 (3.4) **
Trans fat score	7.9 (1.3)	7.9 (1.2)	7.9 (1.4)	7.9 (1.1)	7.8 (1.4)	8.2 (1.3)
Omega-3 fatty acids score	7.2 (3.3)	7.3 (3.4)	7.2 (3.3)	7.2 (3.3)	7.4 (3.4)	6.8 (3.2)
Polyunsaturated fatty acids (PUFA) score	6.1 (2.2)	6.1 (2.3)	6.1 (2.2)	6.3 (1.7)	6.1 (2.4)	5.8 (2.3)
Sodium score	5.2 (3.3)	4.8 (3.4)	5.4 (3.2)	5.1 (3.4)	5.4 (3.2)	5.0 (3.3)
Alcohol score	6.2 (1.8)	7.2 (1.7)	5.8 (6.1) ***	6.5 (2.1)	6.3 (1.8)	5.9 (1.3)
Vegetable ^b^, serv/d	3.7 (2.6)	3.9 (2.8)	3.6 (2.5)	3.0 (1.7)	3.7 (2.8)	4.4 (3.0) *
Whole fruit ^b^, serv/d	1.2 (1.1)	1.1 (1.1)	1.2 (1.1)	0.90 (0.92)	1.2 (1.0)	1.6 (1.4) **
Whole grains ^b^, g/d	52.2 (50.5)	51.3 (54.1)	52.6 (49.0)	40.8 (33.1)	52.5 (49.2)	65.6 (66.4) *
SSB, fruit juice ^b^, serv/d	2.2 (2.1)	2.5 (2.4)	2.0 (1.9)	2.3 (2.2)	2.3 (2.2)	1.8 (1.6)
Nuts and legumes ^b^, serv/d	0.85 (0.73)	0.80 (0.64)	0.88 (0.77)	0.93 (0.80)	0.85 (0.76)	0.77 (0.54)
Red/processed meat ^b^, serv/d	0.98 (0.99)	1.1 (1.0)	0.95 (0.96)	1.2 (1.2)	0.98 (0.95)	0.68 (0.73) **
Trans fat, % of energy	1.2 (0.5)	1.2 (0.4)	1.2 (0.5)	1.3 (0.4)	1.3 (0.5)	1.1 (0.5)
Omega-3 fatty acids, mg/d	330 (331)	331 (312)	331 (340)	274 (222)	392 (396)	261 (250) *
PUFA, % of energy	7.0 (2.0)	6.9 (2.0)	7.0 (2.0)	7.1 (1.6)	7.0 (2.1)	6.7 (2.0)
Sodium, mg/d	4097 (1978)	4416 (2245)	3954 (1834)	4429 (2265)	4146 (1882)	3579 (1727)
Alcohol ^b^, drinks/d	0.24 (0.89)	0.38 (1.4)	0.18 (0.50)	0.35 (1.1)	0.24 (0.95)	0.10 (0.34)

^a^ Values are mean (SD) for the score of the overall Alternate Healthy Eating Index (AHEI) and its individual components, with respective means (SD) of intake below each food group or nutrient score. AHEI score is a range of 0–110 points; each individual score is 0–10 points. Significant differences determined from *t*-test or ANOVA shown as * *p* < 0.01; ** *p* < 0.01; *** *p* < 0.001. ^b^ Serving size equivalents are: ½ cup of vegetables or whole fruit or legumes = 100 g; ½ cup of whole grains = 31 g; 8 oz of sugar sweetened beverages or fruit juice = 250 mL; 1 oz of red/processed meat = ~28 g; one standard drink (wine, beer or liquor) = 14 g of ethanol.

## Supplementary Materials

The following are available online at https://www.mdpi.com/2072-6643/11/7/1598/s1, Table S1: Comparison of baseline characteristics for adults living in Puerto Rico with and without complete and valid food frequency questionnaire data, Table S2: Sociodemographic and lifestyle factors associated with the Alternate Healthy Eating Index in adults living in Puerto Rico, Table S3: Mean (standard deviation) of servings per day of foods in adults living in Puerto Rican, by sex and age.
