# Supplementary material for: Dietary Intake and Its Determinants Among Adults Living in the Metropolitan Area of Puerto Rico
Source: Nutrients. 2019 Jul 14;11(7):1598. doi: 10.3390/nu11071598 (PMC6683066; doi:10.3390/nu11071598)
Supplement: Supplementary file 1 [file nutrients-11-01598-s001.pdf]

Supplement table 1: Comparison of baseline characteristics for adults living in Puerto Rico with and without complete and valid food frequency questionnaire data

	Without FFQ (n=132)	*With FFQ (n=248)*
% Female	59.1	69.0
Age (years)		
30-44	32.6	27.4
45-59	42.4	50.4
60-75	25.0	22.2
Puerto Rican ethnicity, %	85.6	79.4
Marital status		
Married/living with partner	41.0	43.7
Divorced/separated/widowed	21.3	20.7
Single	37.7	35.6
Education		
Less than 12th grade	13.1	11.3
12th grade	36.9	26.3
Some college or higher	50.0	62.4
Household income ($)		
$0-$10,000	66.7	56.9
$10,001-$20,000	16.7	23.2
>$20,000	16.7	19.9
Employment		
Currently employed	29.6	40.3*
Retired/stay-at-home	49.2	47.6
Unemployed	21.2	12.1
Physical activity		
Sedentary	46.6	42.2
Light	25.0	32.9
Moderate/Vigorous	28.4	24.9
Healthy hours sleep /day	44.5	53.9
Sleep difficulties, %	61.0	45.5*
Receives food assistance, %	52.5	50.4
Current smoker, %	26.3	19.7

Shown as percent. FFQ: Food frequency questionnaire.

*Significantly different by FFQ completion at p<0.05

Supplemental table 2: Sociodemographic and lifestyle factors associated with the Alternate Healthy Eating Index in adults living in Puerto Rico

Characteristic	Percent	AHEI	P-value
Sex			
Men	31.0	58.0 (2.1)	0.70
Women	69.0	58.8 (1.9)	
Age (years)			**0.009**
30-44	27.4	53.9 (2.4)	
45-59	50.4	59.2 (1.8)	
60-75	22.2	62.1 (2.5)	
Ethnicity			**0.011**
Puerto Rican	79.4	55.6 (1.7)	
Other	20.6	61.2 (2.4)	
Marital status			**0.037**
Married/living with partner	43.7	56.8 (2.0)	
Divorced/separated/widowe	20.7	56.7 (2.4)	
Single	35.6	61.7 (2.1)	
Education			**0.026**
Less than 12th grade	11.3	55.2 (2.9)	
12th grade	26.3	57.8 (2.2)	
Some college or higher	62.4	62.2 (1.8)	
Household income			0.89
$0-$10,000	56.9	59.9 (1.7)	
$10,001-$20,000	23.1	58.6 (2.3)	
>$20,000	20.0	57.6 (2.8)	
Employment			0.48
Currently employed	40.3	54.5 (2.0)	
Retired/stay-at-home	47.6	59.4 (2.1)	
Unemployed	12.1	56.2 (2.9)	
SNAP food assistance			0.49
No	49.6	57.7 (2.0)	
Yes	50.4	59.1 (2.0)	
Physical activity			**0.051**
Sedentary	42.2	55.9 (2.1)	
Light	32.9	60.7 (2.1)	
Moderate/Vigorous	28.9	58.6 (2.0)	
Sleep hours/day			0.29
≤6 or ≥9 hours/day	46.2	57.5 (1.9)	
7-8 hours/day	53.8	59.3 (2.1)	
Sleep difficulties, %			0.19
Always/ Occasionally	45.5	59.6 (2.0)	
Rarely	54.5	57.2 (1.9)	
Smoking status, %			**0.018**

Never / former smoker	82.6	61.0 (1.6)
Current smoker	17.4	55.7 (2.5)

Shown as mean (standard error) from a multivariable-adjusted general linear model including all variables shown and total energy intake. AHEI score is a range of 0-110 points with higher scores indicative of better diet quality. n=248

Supplemental table 3: Mean (standard deviation) of servings per day of foods in adults living in Puerto Rican, by sex and age

	Servings/day[a]				
	Sex		Age		
**Food group**	Men (n=77)	Women (n=171)	30-45y (n=68)	46-60y (n=125)	61-75y (n=55)
All sugary beverages	2.68 (2.45)	2.15 (1.95)	2.48 (2.23)	2.42 (2.26)	1.87 (1.59)
Juices	1.66 (1.71)	1.4 (1.58)	1.58 (1.84)	1.53 (1.64)	1.23 (1.24)
Sodas and other sugary beverages	1.02 (1.34)	0.76 (0.98)	0.9 (1.07)	0.89 (1.15)	0.64 (1.03)
Sweets and desserts	3.93 (2.97)	3.87 (3.01)	4.83 (3.29)	3.50 (2.42)	3.61 (3.54)**
Dairy	1.86 (1.79)	1.80 (1.41)	1.71 (1.04)	1.85 (1.52)	1.86 (2.03)
Mixed dishes	0.97 (1.01)	0.82 (0.74)	1.05 (0.95)	0.84 (0.77)	0.72 (0.79)
Mixed dishes	0.50 (0.42)	0.40 (0.35)	0.48 (0.38)	0.44 (0.39)	0.34 (0.35)
Soups	0.48 (0.66)	0.42 (0.54)	0.57 (0.69)	0.39 (0.52)	0.37 (0.53)
Starchy vegetables	1.34 (1.63)	0.99 (1.24)	0.85 (0.79)	1.11 (1.46)	1.37 (1.68)
Potatoes	0.86 (1.31)	0.64 (0.85)	0.54 (0.57)	0.71 (0.98)	0.91 (1.42)
Plantains	0.16 (0.19)	0.14 (0.17)	0.13 (0.15)	0.15 (0.18)	0.16 (0.20)
Sweet potato	0.31 (0.46)	0.22 (0.40)	0.19 (0.23)	0.26 (0.50)	0.30 (0.42)
Fast foods	0.52 (0.52)	0.41 (0.45)	0.59 (0.56)	0.41 (0.45)	0.33 (0.38)**
Rice	0.59 (0.51)	0.50 (0.49)	0.50 (0.42)	0.53 (0.49)	0.55 (0.59)
Fats	0.92 (0.64)	0.85 (0.54)	0.82 (0.46)	0.85 (0.56)	0.99 (0.72)
Refined grains	0.52 (0.49)	0.58 (0.46)	0.60 (0.51)	0.56 (0.42)	0.55 (0.54)
Fruit	1.08 (1.05)	1.16 (1.09)	0.85 (0.92)	1.11 (0.95)	1.54 (1.37)**
All vegetables	3.26 (2.57)	3.38 (2.56)	2.72 (1.69)	3.41 (2.80)	3.96 (2.72)*
Yellow vegetables	0.26 (0.34)	0.33 (0.47)	0.21 (0.23)	0.32 (0.50)	0.40 (0.44)*
Green vegetables	0.38 (0.43)	0.41 (0.48)	0.34 (0.35)	0.38 (0.48)	0.53 (0.53)
Tomatoes	0.44 (0.40)	0.44 (0.43)	0.36 (0.33)	0.43 (0.43)	0.54 (0.49)
Other vegetables	2.19 (2.04)	2.20 (1.67)	1.81 (1.19)	2.28 (2.05)	2.49 (1.72)
Processed meats	0.73 (0.80)	0.71 (0.83)	0.93 (1.07)	0.70 (0.72)	0.51 (0.59)*
Eggs	0.84 (1.03)	0.58 (0.66)*	0.52 (0.53)	0.72 (0.93)	0.68 (0.74)

Whole grains	0.54 (0.48)	0.53 (0.47)	0.45 (0.35)	0.55 (0.51)	0.60 (0.51)
Legumes	0.58 (0.48)	0.65 (0.62)	0.64 (0.54)	0.63 (0.63)	0.61 (0.49)
Poultry	0.33 (0.31)	0.31 (0.26)	0.31 (0.26)	0.34 (0.29)	0.29 (0.27)
Fish and seafood	0.36 (0.47)	0.37 (0.48)	0.34 (0.41)	0.44 (0.57)	0.24 (0.25)*
Snacks	0.36 (0.64)	0.44 ().72)	0.57 (0.78)	0.43 (0.77)	0.17 (0.19)**
Red meats	0.33 (0.41)	0.25 (0.32)	0.32 (0.33)	0.30 (0.40)	0.17 (0.22)*
Pasta	0.20 (0.20)	0.21 (0.26)	0.27 (0.22)	0.19 (0.25)	0.17 (0.23)*
Oils	0.33 (0.27)	0.35 (0.36)	0.26 (0.24)	0.34 (0.33)	0.45 (0.42)**
Nuts	0.22 (0.39)	0.23 (0.49)	0.29 (0.60)	0.23 (0.46)	0.15 (0.20)
Alcohol	0.38 (1.41)	0.18 (0.50)	0.35 (1.06)	0.24 (0.95)	0.10 (0.34)
Artificially sweetened beverages	0.09 (0.39)	0.15 (0.44)	0.16 (0.41)	0.15 (0.51)	0.05 (0.15)
Spices and condiments	2.92 (2.11)	3.08 (2.37)	2.88 (2.03)	3.18 (2.47)	2.88 (2.17)
Salt	0.43 (0.39)	0.48 (0.42)	0.42 (0.44)	0.50 (0.40)	0.44 (0.40)
Unsweetened beverages	1.52 (1.52)	1.36 (1.48)	1.27 (1.68)	1.38 (1.26)	1.65 (1.73)
Water	3.65 (3.32)	4.01 (3.44)	3.04 (3.03)	4.22 (3.52)	4.22 (3.43)*

[a]Shown as unadjusted mean (SD). Estimated with SAS PROC RANK. Significant differences determined from t-test or ANOVA shown as *p<0.05; **p<0.01
